# Supplementary material for: Inequalities of visceral leishmaniasis case-fatality in Brazil: A multilevel modeling considering space, time, individual and contextual factors
Source: PLoS Negl Trop Dis. 2021 Jul 1;15(7):e0009567. doi: 10.1371/journal.pntd.0009567 (PMC8279375; doi:10.1371/journal.pntd.0009567)
Supplement: S1 Table — (DOCX) [file pntd.0009567.s001.docx]

S1 Table. Confirmed visceral leishmaniasis cases according to FU, Brazil, 2007 and 2017

| **BRAZILIAN FEDERATED UNITS** | **NOTIFICATION YEAR** | | | | | | | | | | | **Total** |
| --- | --- | --- | --- | --- | --- | --- | --- | --- | --- | --- | --- | --- |
|  | **2007** | **2008** | **2009** | **2010** | **2011** | **2012** | **2013** | **2014** | **2015** | **2016** | **2017** |  |
| Rondônia (RO) | 0 | 0 | 0 | 0 | 0 | 2 | 2 | 0 | 0 | 0 | 0 | 4 |
| Amazonas (AM) | 0 | 3 | 3 | 1 | 1 | 2 | 0 | 1 | 0 | 0 | 0 | 11 |
| Roraima (RR) | 0 | 1 | 3 | 11 | 12 | 10 | 19 | 13 | 15 | 35 | 32 | 151 |
| Pará (PA) | 202 | 226 | 163 | 188 | 269 | 137 | 190 | 139 | 209 | 263 | 449 | 2435 |
| Amapá (AP) | 1 | 1 | 0 | 0 | 0 | 0 | 0 | 0 | 0 | 0 | 0 | 2 |
| Tocantins (TO) | 404 | 427 | 410 | 346 | 515 | 352 | 277 | 166 | 205 | 211 | 244 | 3557 |
| Maranhão (MA) | 247 | 259 | 253 | 239 | 251 | 135 | 332 | 269 | 361 | 519 | 586 | 3451 |
| Piauí (PI) | 233 | 273 | 162 | 174 | 213 | 161 | 169 | 192 | 120 | 131 | 146 | 1974 |
| Ceará (CE) | 454 | 460 | 531 | 456 | 523 | 335 | 398 | 480 | 423 | 283 | 291 | 4634 |
| Rio Grande Norte (RN) | 62 | 79 | 86 | 71 | 109 | 89 | 76 | 89 | 68 | 74 | 70 | 873 |
| Paraíba (PB) | 19 | 31 | 11 | 29 | 29 | 33 | 26 | 44 | 37 | 13 | 29 | 301 |
| Pernambuco (PE) | 63 | 77 | 58 | 50 | 65 | 57 | 62 | 145 | 167 | 107 | 157 | 1008 |
| Alagoas (AL) | 15 | 8 | 9 | 14 | 16 | 19 | 11 | 17 | 19 | 16 | 34 | 178 |
| Sergipe (SE) | 64 | 29 | 41 | 88 | 76 | 56 | 47 | 65 | 66 | 54 | 72 | 658 |
| Bahia (BA) | 192 | 150 | 260 | 331 | 319 | 234 | 263 | 357 | 263 | 140 | 243 | 2752 |
| Minas Gerais (MG) | 385 | 470 | 508 | 518 | 478 | 386 | 319 | 366 | 459 | 539 | 789 | 5217 |
| Espírito Santo (ES) | 0 | 1 | 5 | 1 | 9 | 2 | 0 | 3 | 8 | 16 | 15 | 60 |
| Rio de Janeiro (RJ) | 1 | 0 | 3 | 0 | 4 | 3 | 6 | 5 | 6 | 6 | 16 | 50 |
| São Paulo (SP) | 233 | 261 | 193 | 199 | 211 | 236 | 181 | 172 | 154 | 144 | 169 | 2153 |
| Paraná (PR) | 2 | 4 | 0 | 4 | 1 | 1 | 0 | 2 | 4 | 10 | 4 | 32 |
| Santa Catarina (SC) | 1 | 1 | 0 | 0 | 2 | 2 | 1 | 0 | 0 | 1 | 4 | 12 |
| Rio Grande do Sul (RS) | 0 | 0 | 6 | 2 | 2 | 0 | 3 | 3 | 1 | 2 | 7 | 26 |
| Mato Grosso do Sul (MS) | 196 | 219 | 176 | 204 | 260 | 295 | 227 | 170 | 127 | 115 | 142 | 2131 |
| Mato Grosso (MT) | 21 | 44 | 60 | 54 | 48 | 43 | 27 | 11 | 18 | 13 | 14 | 353 |
| Goiás (GO) | 15 | 26 | 18 | 24 | 23 | 21 | 30 | 34 | 34 | 31 | 44 | 300 |
| Distrito Federal (DF) | 36 | 43 | 41 | 27 | 33 | 28 | 40 | 36 | 36 | 31 | 49 | 400 |
| VL confirmed cases | 3565 | 3991 | 3894 | 3704 | 4107 | 3269 | 3472 | 3733 | 3558 | 3455 | 4456 | 41204 |
| VL cases confirmed and with clinical outcome known | 2846 | 3093 | 3000 | 3031 | 3469 | 2639 | 2706 | 2779 | 2800 | 2754 | 3606 | 32723 |
