# Supplementary material for: Inequalities of visceral leishmaniasis case-fatality in Brazil: A multilevel modeling considering space, time, individual and contextual factors
Source: PLoS Negl Trop Dis. 2021 Jul 1;15(7):e0009567. doi: 10.1371/journal.pntd.0009567 (PMC8279375; doi:10.1371/journal.pntd.0009567)
Supplement: S2 Table — (DOCX) [file pntd.0009567.s002.docx]

S2 Table. VL incidence by FU, between 2007 and 2017

|  | 2007 | 2008 | 2009 | 2010 | 2011 | 2012 | 2013 | 2014 | 2015 | 2015 | 2016 |
| --- | --- | --- | --- | --- | --- | --- | --- | --- | --- | --- | --- |
| RO | 0,19 | 0,00 | 0,00 | 0,00 | 0,06 | 0,13 | 0,12 | 0,00 | 0,00 | 0,00 | 0,00 |
| AM | 0,03 | 0,12 | 0,09 | 0,03 | 0,03 | 0,06 | 0,00 | 0,05 | 0,00 | 0,03 | 0,00 |
| RR | 0,72 | 0,48 | 1,66 | 3,77 | 3,48 | 2,34 | 4,28 | 3,59 | 4,09 | 8,18 | 6,77 |
| PA | 5,10 | 5,00 | 4,05 | 4,12 | 4,75 | 3,17 | 3,13 | 2,82 | 3,26 | 4,10 | 6,68 |
| AP | 0,16 | 0,16 | 0,00 | 0,00 | 0,00 | 0,00 | 0,00 | 0,00 | 0,00 | 0,13 | 0,00 |
| TO | 31,20 | 38,11 | 35,91 | 26,89 | 37,05 | 26,17 | 20,24 | 12,39 | 14,37 | 15,46 | 16,98 |
| MA | 4,80 | 6,41 | 5,81 | 5,38 | 5,73 | 3,43 | 7,59 | 6,28 | 7,50 | 9,35 | 10,39 |
| PI | 11,84 | 14,49 | 8,49 | 7,95 | 9,97 | 9,36 | 12,36 | 12,92 | 9,79 | 8,07 | 9,34 |
| CE | 6,60 | 6,58 | 7,92 | 6,40 | 7,16 | 4,76 | 5,47 | 7,02 | 5,73 | 4,05 | 4,34 |
| RN | 2,30 | 2,99 | 3,06 | 2,65 | 3,78 | 2,97 | 2,40 | 2,94 | 2,42 | 2,54 | 2,78 |
| PB | 0,68 | 1,10 | 0,56 | 0,88 | 1,11 | 1,05 | 0,95 | 1,51 | 1,17 | 0,83 | 1,18 |
| PE | 0,88 | 0,97 | 0,94 | 0,76 | 0,96 | 0,78 | 0,78 | 1,85 | 1,94 | 1,24 | 2,01 |
| AL | 1,04 | 0,80 | 0,98 | 1,09 | 1,18 | 1,17 | 0,77 | 1,32 | 1,34 | 0,79 | 1,42 |
| SE | 3,69 | 2,00 | 2,23 | 4,35 | 3,73 | 2,70 | 2,30 | 3,05 | 3,11 | 2,46 | 3,41 |
| BA | 1,65 | 1,36 | 2,38 | 2,81 | 2,68 | 2,12 | 2,22 | 3,52 | 2,60 | 1,61 | 2,17 |
| MG | 2,15 | 2,65 | 2,89 | 2,96 | 2,56 | 2,02 | 1,66 | 1,90 | 2,29 | 2,67 | 4,12 |
| ES | 0,00 | 0,09 | 0,23 | 0,06 | 0,31 | 0,06 | 0,11 | 0,11 | 0,21 | 0,46 | 0,61 |
| RJ | 0,02 | 0,00 | 0,04 | 0,01 | 0,03 | 0,03 | 0,05 | 0,04 | 0,04 | 0,05 | 0,11 |
| SP | 0,63 | 0,75 | 0,56 | 0,55 | 0,56 | 0,61 | 0,47 | 0,44 | 0,40 | 0,39 | 0,43 |
| PR | 0,03 | 0,04 | 0,01 | 0,05 | 0,02 | 0,05 | 0,00 | 0,02 | 0,05 | 0,12 | 0,04 |
| SC | 0,02 | 0,02 | 0,00 | 0,00 | 0,03 | 0,03 | 0,02 | 0,00 | 0,00 | 0,03 | 0,06 |
| RS | 0,00 | 0,00 | 0,08 | 0,02 | 0,02 | 0,00 | 0,03 | 0,04 | 0,01 | 0,02 | 0,07 |
| MS | 10,12 | 10,79 | 8,30 | 8,78 | 11,02 | 12,37 | 9,46 | 6,79 | 5,01 | 4,73 | 5,56 |
| MT | 1,07 | 1,86 | 2,27 | 1,81 | 1,79 | 1,73 | 1,08 | 0,55 | 0,75 | 0,45 | 0,62 |
| GO | 0,33 | 0,48 | 0,39 | 0,55 | 0,48 | 0,42 | 0,50 | 0,61 | 0,57 | 0,58 | 0,76 |
| DF | 2,34 | 2,39 | 2,26 | 1,44 | 1,53 | 1,40 | 1,63 | 1,57 | 1,33 | 1,76 | 2,01 |

Brazilian Federated Units: Rondônia (RO), Amazonas (AM), Roraima (RR), Pará (PA), Amapá (AP), Tocantins (TO), Maranhão (MA), Piauí (PI), Ceará (CE), Rio Grande Norte (RN), Paraíba (PB), Pernambuco (PE), Alagoas (AL), Sergipe (SE), Bahia (BA), Minas Gerais (MG), Espírito Santo (ES), Rio de Janeiro (RJ), São Paulo (SP), Paraná (PR), Santa Catarina (SC), Rio Grande do Sul (RS), Mato Grosso do Sul (MS), Mato Grosso (MT), Goiás (GO), Distrito Federal (DF)
