# Supplementary material for: Inequalities of visceral leishmaniasis case-fatality in Brazil: A multilevel modeling considering space, time, individual and contextual factors
Source: PLoS Negl Trop Dis. 2021 Jul 1;15(7):e0009567. doi: 10.1371/journal.pntd.0009567 (PMC8279375; doi:10.1371/journal.pntd.0009567)

Preliminaries

```
library(mgcv, warn.conflicts = FALSE)
library(dplyr, warn.conflicts = FALSE)

## inverse logit function
logit_inv <- function(a) {
  exp(a) / (exp(a) + 1)
}
```

Generate fake data

```
## create region-level data
set.seed(513)                                 # for reproducibility
x <- runif(20, 0, 8)                          # predictor variable
y_mu <- logit_inv(-2 + -0.1 * x^2 + 1 * x)  # mean CFR for each region
dat_region <- data.frame(region = seq_along(x), x, y_mu)

## simulate patient outcomes, 20 patients per region
dat_list <- list()

for (i in 1:nrow(dat_region)) { # for each region...
  dat_list[[i]] <- data.frame(
    region = dat_region$region[i],
    x = dat_region$x[i],
    outcome = rbinom(20, 1, dat_region$y_mu[i])
  )
}

## bind list into single data frame
dat <- do.call(rbind.data.frame, dat_list)
```

Summarize observed case fatality (including confidence intervals) by region

```
dat_plot <- dat %>%
  group_by(region, x) %>%
  summarize(
    cfr_mean = mean(outcome),
    cfr_low = binom.test(x = sum(outcome), n = length(outcome))$conf.int[1],
    cfr_upp = binom.test(x = sum(outcome), n = length(outcome))$conf.int[2],
    .groups = "drop"
  )
```

Fit GAMM model

```
mod <- gamm(
  outcome ~ s(x),
  random = list(region = ~1), # random intercepts by region
  data = dat,
  family = "binomial"
)
```

```
##
##  Maximum number of PQL iterations:  20
```

Plot GAMM fit plus region-specific CFR estimates

```
plot(
  mod$gam,
  shift = coef(mod$gam)[[1]], # un-center (add model intercept)
  trans = logit_inv,      # transform to probability scale using inverse logit
  ylim = c(0, 1),
  rug = FALSE,
  ylab = "Case fatality"
)
points(cfr_mean ~ x, data = dat_plot)
segments(dat_plot$x, dat_plot$cfr_low, dat_plot$x, dat_plot$cfr_upp)
```

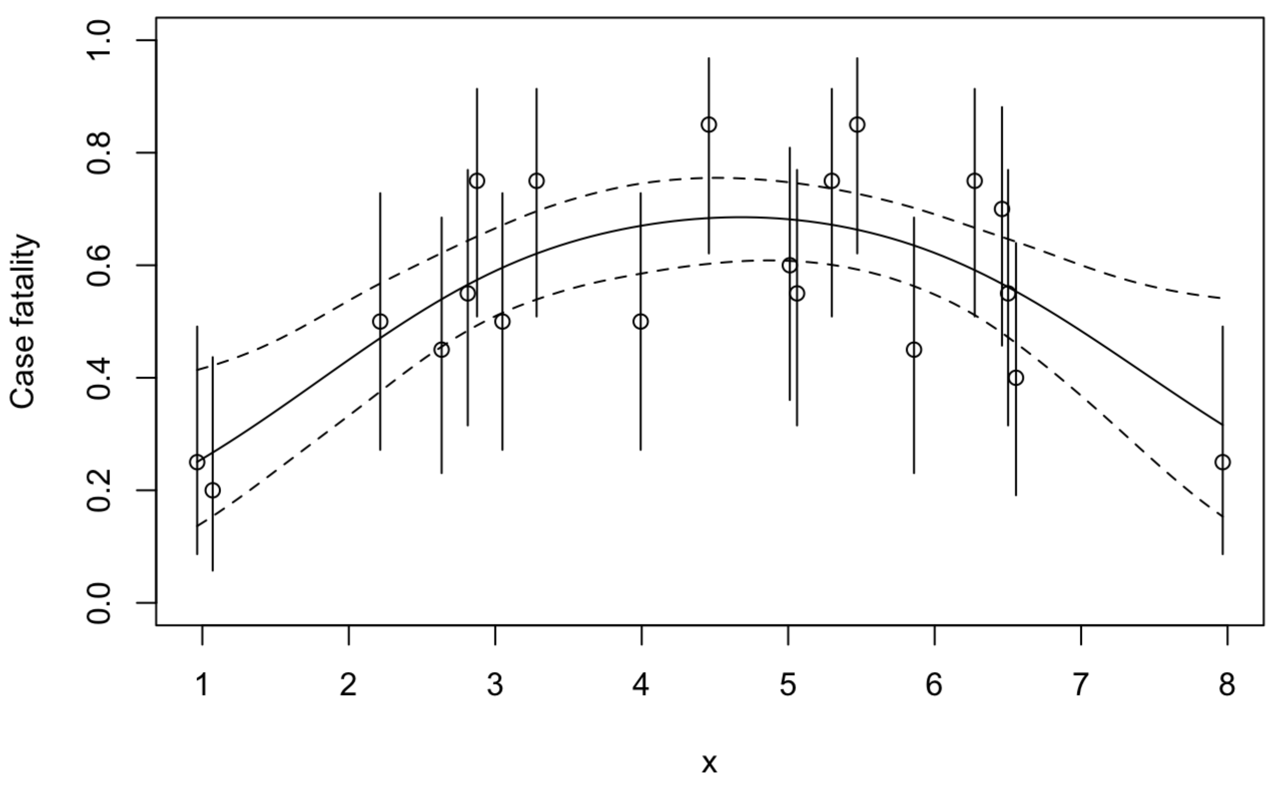

Supplement: Attachment — Submitted filename: gam_plot_example.pdf [file pntd.0009567.s005.pdf]
