# Supplementary material for: Inequalities of visceral leishmaniasis case-fatality in Brazil: A multilevel modeling considering space, time, individual and contextual factors
Source: PLoS Negl Trop Dis. 2021 Jul 1;15(7):e0009567. doi: 10.1371/journal.pntd.0009567 (PMC8279375; doi:10.1371/journal.pntd.0009567)
Supplement: Attachment — Submitted filename: answer to reviewers.docx [file pntd.0009567.s003.docx]

January 4, 2021

Alberto Novaes Ramos Jr, M.D., M.P.H., Ph.D.
Guest Editor
PLOS Neglected Tropical Diseases

Manuscript: PNTD-D-20-01885
"Inequalities of visceral leishmaniasis case-fatality in Brazil: a multilevel modeling considering space, time, individual and contextual factors"

Dear Dr. Alberto Novaes

Thank you for handling and considering our submission to *PloS Neglected Tropical Diseases*. We are also grateful to the reviewers for their time and valuable contributions to improve the manuscript.

We have carefully considered all reviewers concerns and did our best to improve the text accordingly. In all cases we’ve included in the manuscript the required explanations. The modifications implemented in this revised version are summarized below together with specific answers to the questions placed by the reviewers.

Please let us know in case any other improvement in the manuscript becomes necessary.

Sincerely,

Gláucia Cota.

**Answer Letter to Reviewers**

Reviewer #1: Adequate methods have been used in the analyses.
1. However, the original data (national) seems to be of low quality, particularly those regarding the clinical data. Often, non-qualified personnel extracts the data from the clinical files in health units. It is noteworthy the lousy quality of the clinical descriptions by physicians in the country. The original data's low quality may have hampered the data analyses and reduced the conclusions' credibility. The authors should address this point in the discussion.

We fully agree with the reviewer. This point is now discussed in the manuscript.

2. It might also be better to standardize the proportion of deaths among the patients with the disease since the authors used both case-fatality (more appropriate) and lethality. We have standardized case-fatality along the manuscript

3. Figures are hard to be read due to the low resolution, and there is no apparent correspondence of the figures with their legend. This fact has hampered the review quality.

New and higher quality figures were made.

4. The authors should be clear that the policy changes to VL's diagnosis and treatment in Brazil have worsened case-fatality and not use the euphemistic wording that they had not improved it. Possible explanations are that patients diagnosed by rapid tests are usually at the primary care units. There, the use of antimonials is much more likely due to the national recommendations themselves, and severe cases may not be identified and forwarded to secondary- or tertiary units and not adequately treated. Another point is that any clinical trials have not evaluated the treatment of severe cases of VL up to date. For the paper quality, improvement in data interpretation and conclusions are necessary. This is a relevant and well-done study that should be published. However, changes in the discussion and conclusions seem to be appropriate.

Thank you for your comments. We agree that the policy changes proposed by the Brazilian Minister of Health were insufficient insofar as they were slow, timid and had their implementation poorly planned. These points were added to the discussion.

Reviewer #2:

-  Abstract: several findings are only mentioned first in the conclusion, would recommend to put these in the “Findings”. The abstract was re written

-       summary: In the abstract hospital beds is mentioned as factor but not in the summary. This finding was added to the summary
-       line 62: “trends and factors – recommend to add “and” It was added
-       line 126: justified: suggest to replace by “explained” – also in the discussion. It was replaced
-       line 347: in contrast to another: suggest to replace “another” by “the other” It was replaced
-       line +516: lack of diagnostic confirmation: does it mean not test was done or that the test could be negative but the patient was still diagnosed on clinical grounds? The sentence was clarified: “The fatal outcome related to lack of VL laboratorial confirmation, in other words, VL diagnosed on clinical grounds…”

This is a useful paper on an important topic. The data are clearly presented, the analysis was conducted carefully and the findings are nicely discussed. I only have a few minor points – all discretionary. All suggestions were accepted, and corrections made in the manuscript.

Reviewer #3: I found the objectives to be clearly stated in the Abstract (lines 29-31), but suggest minor revision to more clearly restate/elaborate at the end of the Intro (127-131). The end of the Introduction section was restated to clarify the objectives of this study.

The study design with respect to the temporal trend and spatial dependency in case fatality is generally strong, and I have only minor methodological suggestions. But with respect to assessing determinants of lethality at the individual and regional level, I found the methodology (and results) to be incompletely described or ambiguous in quite a few places. When I read the Methods section on its own I had only small suggestions/questions. But when I read the Results I had substantial difficulty mapping stated results back to described Methods. I'll describe most of these issues later in the Results section. We have improved the methods description in order to make the analyses performed and results clearer. The changes in the manuscript are presented below, following the reviewer suggestion always whenever possible.

1. The distinction between "federated unit (FU)" and "FU-by-year" groupings should be clarified. Throughout the text there are references to two categories: FU with below-median case fatality and FU with above-median case fatality. But at a few points in the text it seems like the actual split for analysis purposes was 'FU-by-year combinations below the national median' vs. 'FU-by-year combinations above the national median'. E.g. Line 360: "Some FUs can appear in both groups in Fig 3 since the FU's position in relation to national lethality, in some cases, was not stable over the period 2007-2017." (In fact, I count 18/26 FU in Fig. 3 that appear in both the above-median and below-median groups). I don't think it's necessary to split FU into groups at all (see #2), but if the split is retained I think it would be preferable to actually split by FU (based on FU-specific fatality across all study years) rather than FU-by-year. The latter (i.e. current) approach might be prone to statistical artifacts due to varying year coverage for different FU within each model.
Thank you for the opportunity to clarify this point. In the current paper version, we have accepted the reviewer suggestion and the analyzes were performed without dividing groups by the median of VL case fatality. The models were adjusted for the database (all cases), leaving the random effect of the intercept (baseline risk varying between UF's) to make this distinction for itself in the model. The term temporal was included in the model as a covariate, after the temporal analysis, which was done through the GAM model. It pointed to a linear effect of time. In addition to explicitly considering the increasing temporal trend of VL case fatality, this term captured the serial correlation between the residuals of the generalized mixed linear model. During the analysis of individual dataset, we did not explore the FU-year interaction, instead of, time was taken as a fixed variable and the variability between UFs evaluated through a random intercept at this level of aggregation. The interaction between these terms has not been analyzed. The methodology and results have been rewritten according to this new methodology.

2. I don't see any reason to analyze the below-median FU and above-median FU using separate models (or perhaps below-median and above-median *FU-years*, see above) (e.g. lines 218-225). It's of course possible that some factors have non-linear effects on case fatality, but I can't think of a priori reasons for such effects in most of the variables analyzed here, and there's little relevant discussion in the manuscript. Indeed the reported odds ratios are almost always similar between the above-median and below-median groups (Table 3). Splitting on the median seems arbitrary and could have undesirable side effects:

- Halving the sample size in each model reduces the statistical power to detect 'real effects'
- Using two models instead of one increases the likelihood of false-positives, particularly given the 'forward selection on many variables' approach used here.

Perhaps the biggest challenge is that it's not clear what to make of results that apparently differ between the two categories (e.g. HIV-coinfection, education, and hospital beds/capita are suggested to only affect cases fatality in above-median FU). Is the lack of a significant result in the below-median group a false negative due to lack of statistical power? Or the above-median result a false positive? Or does the effect of these variables on fatality really change as a function of FU-specific fatality levels? I think a single-model approach would be preferable here, and much simpler to present and interpret. If the effect of any given variable on case fatality appears non-linear, it could be modelled using e.g. a polynomial term, a GAM smoother, or investigated using supplementary analysis on FU subsets.

As we informed in the previous question, the reviewer's suggestion was accepted and the analyzes carried out again considering all cases together. The analytical approach presented in the first version of this manuscript aimed to identify differences between subgroups, which are in fact discreet. We agree that the most relevant information in this analysis is the confirmation of the presence of a random effect confirming that other variables at FU level are important, but which have not been measured. Among the possible limiting factors, there is the analysis scale, focused here on the UF, which may have softened an effect of greater magnitude at the municipal level.

3. Minor suggestions relating to Methodology:

188: "adjusted" is not quite right here. Suggest using something like "generalized additive models (GAMs) were fit..." instead. The sentence was rewritten.

188: Is it necessary to use a GAM rather than the simpler GLM here? The time-series in Fig 1 looks reasonably linear to me, and the straight GAM fit line (Fig 1 right) seems to indicate zero smoothing. Also, the single temporal coefficient presented in the results (4% per year, line 277) seems to represent a GLM coefficient rather than a GAM coefficient, no? If sticking with GAM, clarify the smoother type (thin plate, penalized cubic, etc.).

Yes, we think so. We have used GAM precisely to assess the effect of time on lethality, whether linear or non-linear. Detecting the linear effect, in the following linear modeling, at the individual level, a fixed linear effect of time (year of notification) was considered and the smoother type was informed.

196-199: If the ultimate CAR model uses counts of deaths as the response and counts of cases as an offset, the Empirical Bayes smoothing seems unnecessary to me. I.e. The higher uncertainty in FU with low case counts is already explicitly incorporated into the CAR model with the offset term. If retained, the Empirical Bayes methods should be described in more detail (software used, model structure, settings, etc.).

The empirical Bayesian rates were made as a previous analysis to the CAR model, in a descriptive mapping of the lethality, but we agree that it is unnecessary, and we removed it from the manuscript.

201-204: Suggest clarifying the structure of the non-spatial term. Random intercepts?

Yes, random intercepts. It was clarified in the text.

205-207: Suggest rephrasing to clarify that the offset is the number of confirmed VL cases. Also, don't need to put "offset" in quotes here as the term has already been used at line 189.

Ok, the sentence was rephrased.

214: Clarify the difference between the "random term at the FU level" and the "random intercept at the FU level" described in the previous sentence. Does the "random term" mean a 'random slope' term for each FU with respect to year or some other variable?

Thank you for the opportunity to make it clearer. It was clarified in the manuscript.

227: Clarify which "parametric or non-parametric hypothesis tests" were used, the software, etc. Also, are these results presented anywhere (perhaps these are the 'Univariate' results in Table 3?). Otherwise it's not clear what the purposes of these tests was.

We have added the tests and softwares used in Methods section. The tests were used in the selection of candidate variables to be part of the multiple model, and in the analysis of multicollinearity (evaluation of correlation between explanatory variables).

229: Clarify how multicollinearity was tested for, which variables were affected, and what actions were taken when it was observed.

This information was added: “*The associations between each variable with outcome (death from VL) and among the explanatory variables - as a previous strategy for detecting possible multicollinearity in multiple models, were explored by parametric or non-parametric hypothesis tests (tests for proportions - chi-square test; test of means or medians - t-test or Wilcoxon, analysis of variance or Kruskal-Wallis, and correlation coefficients - Pearson and Spearman, depending on the data's distribution pattern). When a high correlation between two explanatory variables (> 85%) was detected, those with the greatest association with the outcome were selected for the multiple models [35]. “*

248: This line indicates that analyses were done in R and SPSS, but apart from the CAR model (Line 209) it's not clear which software/packages were used for which statistical analyses.

CAR was also done in software R through the INLA package. this information was added to the text.

As noted above, particularly with respect to the analyses on determinants of lethality at the individual and regional level, I had substantial difficulty linking the stated results back to the described Methods.
1. It's not clear to me what "Univariate analysis" and "Multiple analysis" refer to (e.g. Table 3). Normally I would think "Univariate" refers to independent tests between fatality and each separate variable (whereas "Multiple" refers to the multivariate models obtained through forward variable selection, i.e. 232-248), but I don't see the univariate tests described anywhere in the Methods. Also, lines 244-246 make me think random coefficients are a final step in the forward selection (i.e. "Multiple analysis") process, but then there is a random-coefficient term in the Univariate column of Table 3 (though based on Lines 343-345 perhaps this term was placed in the wrong column?).
There's also not enough description of the apparent difference between Univariate/Multiple results. What does it mean that the best Multiple model for above-average FU contained only a "Report year" term and no other variables? And that the odds ratios for "Report year" are below 1 in the Multiple analysis but above 1 in the Univariate?

The reviewer interpretation was correct, we use univariate analysis to explore each variable separately and, in this model, we were able to estimate the crude OR (unadjusted). The random intercept and time were included in all models, both in univariate and in multiple models, since we had detected in the spatial analysis (CAR), performed a priori, a significant unstructured random effect (no related to the UF neighborhood), and a linear effect of time in GAM. In the modeling procedure, after defining which variables that would remain in the multiple model, and how they would enter (categorized or truncated) using GAMM, we have tested the effects of the random coefficients among these contextual variables (effect of each variable was not estimated only as an average effect on the territory, the effect on the outcome varied among FUs). The method session was presented in details and the results changed with the new analysis methodology proposed by the reviewer, without separating it into two groups of FUs.

2. It's not always clear how the stated results relate to the presented statistics. E.g. Why are HIV-coinfection and education level stated to be linked to case fatality only in above-median FU (337-339; also noted in the Abstract and Discussion)? In Table 3 the relevant odds ratios look similar for both the above-median and below-median groups. Similarly, the Discussion (520-522) notes the absence of an effect of 'hospital-beds/capita' on case fatality, but again it's not clear to me where this result comes from given the similar odds ratios in Table 3.

The division of cases into two groups of FUs according to case fatality median was abolished in this revised version of the manuscript. Now, the univariate and multiple analysis are presented in separate tables and the methods used in each analysis step are detailed.

3. Lines 343-345, 354-356, 356-359: I think the results relating to random coefficient terms are generally misinterpreted (also in the Abstract). The 'hospital beds/capita' term seems to be a 'random coefficient/slope' with respect to FU (i.e. Fig 3 left). In that case, the inclusion of this term through variable selection indicates that the slope of the relationship between 'hospital beds/capita' and case fatality varies by FU. E.g. Based on Fig 3, in federated unit "AL" the slope of the relationship between 'hospital beds/capita' and case fatality is relatively more negative than the average (i.e. the fixed effect) and in "MA" the slope of the relationship between 'hospital beds/capita' and case fatality is relatively more positive than the average.

With the new modeling, the results have changed and were rewritten. But the interpretation of the hospital beds effect made by the reviewer is correct, the effect of this variable on lethality depends on the FU.

4. Lines 363-365: Suggest rephrasing. The lack of a significant FU effect in the low-lethality group doesn't necessarily mean "case fatality can be explained by factors at the individual level". Unexplained variation in the model could be occurring at other levels (i.e. any level between individual and national except FU). We agree. The sentence was suppressed.

5. For each model selection process, suggest clarifying in the Results which variables made it into the final model. Is it all the variables in Table 3 with non-missing odds ratios? Or are the odds ratios given only for variables with p < 0.05? Relatedly, it's not clear to me why there aren't odds ratios given for every variable in the Univariate columns (perhaps related to #1). Also need to better explain the process for dealing with multicolinearity (229-230, 241-243), and which (if any) variables were de-selected as a result. Surely some of the variables in Table 3 are highly correlated, no?
Table 2 shows only the univariate analysis for variables with a linear association with the outcome death from VL. The multicollinearity analysis methodology has been described in methods, besides that, the Figure 5 with this analysis was included.

6. Clarify levels/units of each variable (e.g. Table 3). For instance, giving an odds-ratio for "Gender" doesn't indicate which direction the effect is in. And what are the possible values of e.g. "Education level"? The single odds ratio makes it seem like it's a continuous variable here?
In the revised version of the manuscript the units of the variables are presented, as well as the direction of the effect: for each comparison, the base condition was indicated.

7. Make sure to report the complete test results for statistical tests (usually test statistic, degrees of freedom, and p-value). E.g. Table 2 (Results) gives bare p-values and I can't find any details on test type within the text.

The complete description of the statistical tests has now been included in the method section.

8. Lines 339-341, 354-356: Clarify the metric being used to compare the relevant influence of different variables.

The complete description of the statistical tests has now been included in the method section: *“The contribution of each added variable was tested at by testing the likelihood ratio between nested models or by the Akaike Information Criterium - AIC for non-nested models.”*

9. Make sure figure axes have meaningful labels. E.g.
- Fig. 1 right, y-axis: "Case fatality (logit scale)" (or transform to probability scale)
- Fig. 2 middle, x-axes: "Federated unit (integer index)"
- Fig. 3, x-axes: "Estimated model coefficient (case fatality on logit scale)"

All figures were performed again and completed with the necessary information.

278: Clarify the time unit. 4% increase per year or over the 11-year period? Would be good to add confidence interval for the trend estimate here too.

This information was clarified. It was an average increase of case fatality of 4% for each year. The confidence interval (CI95%: 2,5 – 4,9%) was also added.

286-290, 301-304: The caption and in text-references to the middle panel of Fig 2 are reversed compared to the figure, which shows uf.nu on the left and uf.theta on the right. The caption and in text-references were corrected.

292-293: Clarify the evidence for "good fit regarding the estimates of hyperparameter precision".

This information was added to the manuscript: “*The inference on the parameters and hyperparameters were approximated by the deterministic procedure Integrated Nested Laplace Approximations (INLA) [33]. In sequence, the appropriateness was verified by analyzing the significance of the hyperparameters, through the posterior probability distribution, besides comparing the number of deaths observed with the number of deaths estimated by the model through the map.”*

296: The use of "adjusted" is a bit confusing here. Would be clearer to say something like "Based on this result, we included FU-level random intercepts in our multi-level models of lethality described in the following section". The suggestion was accepted

297-299: This line on temporal effects seems out of place in the spatial section. Suggest moving to temporal section instead. The suggestion was accepted

- 79: what's the time period over which the 200k-400k cases were added? The information was added: *“estimated new Visceral leishmaniasis (VL) case annually”*

- 97: consider "Americas region" instead for clarity The suggestion was accepted

- 121/126: consider "explained" instead of "justified" The suggestion was accepted

- 149: "accessed" instead of "assessed" The suggestion was accepted

- 162: more common to describe Methods in past tense ("will also be considered" -> "were also considered") The suggestion was accepted

- 340: consider "influential" instead of "influent" The suggestion was accepted

- Table 1 (Methods): consider reference/footnote for race categories (e.g. IBGE), as terminology might vary by country The suggestion was accepted

- Table 2 (Results): "percent" instead of "percentual" The suggestion was accepted

- Table 3 (Results): use "." as decimal separator for consistency with rest of manuscript The suggestion was accepted

I think the study objectives are important, and the data sources used are well-suited to addressing them. The analyses with respect to temporal trend and spatial dependency in case fatality from visceral leishmaniasis are generally sound and well-presented. However, there are major shortcomings in the description of the methodology and results for the analyses on individual- and regional-level factors impacting case fatality, which make it difficult to assess the quality of those analyses and interpretation of results. We hope to have clarified these shortcomings in methods/results sections accordingly.
