# Supplementary material for: Inequalities of visceral leishmaniasis case-fatality in Brazil: A multilevel modeling considering space, time, individual and contextual factors
Source: PLoS Negl Trop Dis. 2021 Jul 1;15(7):e0009567. doi: 10.1371/journal.pntd.0009567 (PMC8279375; doi:10.1371/journal.pntd.0009567)
Supplement: Attachment — Submitted filename: answer to reviewers_secondReview.docx [file pntd.0009567.s004.docx]

April 4, 2021

Alberto Novaes Ramos Jr, M.D., M.P.H., Ph.D.
Guest Editor
PLOS Neglected Tropical Diseases

Manuscript: PNTD-D-20-01885
"Inequalities of visceral leishmaniasis case-fatality in Brazil: a multilevel modeling considering space, time, individual and contextual factors"

Dear Dr. Alberto Novaes

Again, we thank the reviewers. We are grateful for the opportunity to discuss in depth our results and methodological choices. Certainly, this process, which for us was rich in learning, will make the manuscript even clearer and more robust. All modifications implemented in this revised version are summarized below together with specific answers to the questions placed by the reviewers.

Please let us know in case any other improvement in the manuscript becomes necessary.

Sincerely,

Gláucia Cota.

**Answer Letter to Reviewers**

Reviewer #1: Reviewer #1: Methods are excellent, well designed and well applied. Very relevant results, likely influencing policy making. Conclusions are in accordance with the results, albeit debatable. Just change the word case-lethality for case-fatality in line 66. Minor revision. *Thank you for this comment. The correction was performed.*

Reviewer #3: Most of my comments related to methodology from the previous round of revision have been addressed with this revised version. However, I have some new concerns regarding the new analyses.

1. Much like the approach of splitting FU into below-median and above-median groups in the original analysis, the approach of discretizing the continuous variables in this revised version seems arbitrary and subjective, and the methodology is not defined clearly enough to be reproducible. E.g. For variable 'Age (years)', why is there no split at the change-point around age 10 (Fig. 5b)? Regardless of how the split-points are chosen, the process of discretizing continuous variables seems unnecessary, and reduces the information available. E.g. The discretized analysis with age only tells us that the >=20 group experiences higher CFR on average than the <20 group, whereas if we retain age as a continuous variable we see that CFR closely mirrors the typical 'bathtub-shape' human mortality curve — decreasing from birth to age ~10, then progressively increasing with age thereafter.
*The process of categorizing or using filters on variables was not arbitrary, but based on the interpretation of the GAM models.* *The GAM model consists of a graphical (visual) analysis methodology that allows the identification of association between explanatory and outcome variables, which can be declared when the value observed does not belong to the estimated confidence interval including zero value. If the association is significant, we can also infer about the direction of the association, whether it is positive or negative. It is important to note that GAMM does not effectively measure the mean effect (risk or odds ratio) of the explanatory variable on the outcome, it just adjusts the local effect by the explanatory variable using splines. There are countless ways to apply the GAM models for this purpose, including: categorizing the variable using the curve inflection points (points of change in the direction of growth and decrease) and to analyze such variable including only the values contained in the value ranges with a significant association with the outcome (values included in the confidence intervals that do not contain zero) – we call it “truncation”. Another option is to dichotomize the original variable at the cutoff point whose direction of association with the outcome changes and subsequently to analyze using linear models in the ranges of values that are linear or, alternatively, between the categories created. That what was done for “age” in this study: below 20 years, there is a protective effect (Odds <1) and above 20 years, it is a factor risk (Odds> 1). To clarify this point, GAMM approach was explained in detail in the methods section. Please, see below the example of the age variable, which presented a change in the effect direction on the curve*. *Age was dichotomized at <20 (inverse effect with the outcome) and> = 20 (direct relationship with the outcome), in order to effectively measure the effect on the GLMM, to facilitate interpretation and not to violate the assumption of a linear relationship with the outcome. It is important to note that, in this case (age variable), if we adjust the variable as continuous, the observed effect was less than that seen with the categorization of the variable.*

2. Likewise, the discretized analysis of onset to reporting delay suggests that CFR is ~6x higher in the >=200-day group compared to the <200-day group, but it's not clear how meaningful this is considering that the 200-day split point is extremely high in the distribution of reporting delay (97th percentile based on data in Table 2). It's hard to tell without seeing the raw data, but the spline relationship between CFR and reporting delay looks relatively linear, in which case it would be more meaningful to interpret a linear coefficient (e.g. X% increase in CFR for every additional week of delay).
*Just like the previous question, explanation for this observation involves the approach based on the GAM model. The relationship between “time to reporting” and case-fatality is not linear, as you can see in figure below, based on GAM model. There is a point of change in the correlation trend. Probably due to this, assuming it as homogeneously linear – what is technically incorrect, the estimated effect was extremely low. On the other hand, using the GAM model and splitting this variable into two ranges, it was possible to measure the effect on the GLMM with the assumption of linearity with the outcome.*

3. Instead of the discretized approach, you could simply use linear terms for the continuous variables, perhaps using transformations (log, square-root, etc.) for variables where the linearity assumptions are severely violated.

*The observation about the possible non-linearity relationship between the variables and the outcome was first mentioned by the reviewer - and it seemed very pertinent to us. For this reason, the GAM models were used to guide the truncation or dichotomization of continuous variables in order to guarantee the linearity premise before evaluating the association with the outcome. This strategy allowed us to work with the variables without applying transformations, which is preferable. Finally, in addition to a predictive model, we opted for explanatory models involving the outcome of interest.*

4. Alternatively, you could use splines for all analyses. E.g. For the univariate analyses you could compare a spline on the variable of interest (x) to the appropriate null model, using likelihood ratio tests or AIC, e.g.

m1 <- gam(outcome ~ s(x) + year + s(FU, bs = "re"), family = "binomial")

m0 <- gam(outcome ~ year + s(FU, bs = "re"), family = "binomial")

anova(m1, m0, test = "LRT")

Admittedly this approach could be difficult for the multivariate analysis, as complex spline models with many variables might not converge.

*Exactly. This analysis was done for all variables. Note that evaluating the individual significance of a variable in the univariate model corresponds to doing the likelihood ratio test (or using the Akaike Information* *Criterium -AIC) comparing the models (null model + s (variable)) and the null model.*

5. Regardless of the approach, I would strongly suggest adding the actual data points to plots of CFR vs explanatory variables (e.g. Fig 5), so that readers can assess the underlying distributions/relationships.
*The figures were built in the R package, which unfortunately does not allow configuration changes. The title of the Y axis was adequate to make the figure more intelligible.*

6. There are methodological aspects of the multilevel modelling that are still unclear, and I'm not confident that another author with the same data could reproduce this analysis without further information. E.g. 250: The use of "subsequently" is confusing here. Is this describing the multilevel modelling, and suggesting that FU-level variables were tested and added prior to individual-level variables? Presumably this is not the case, given that no FU-level variables made it into the final model (Table 3).

*Thanks for the opportunity to clarify: the variables at FU level were first introduced and after them, variables at individual level were included, a sequence respecting only the order of individual significance. Observe that it is not a hierarchical model, in which even though the variables lose their significance with the entry of other variables, they remain in the model. Different from a hierarchical model, in this case, variables that lost statistical significance were removed to obtain a model with a better fit. We have rewritten the methodological aspects of the multilevel modelling* *and* *removed from the text terms that could allude to hierarchical modeling.*

7. 259-260: This line indicates an alpha = 20% acceptance threshold for "variables at the individual level", but what about the FU-level variables? Do they have a different threshold?
*The significance level of 20% was used in the univariate analysis, both for variables at the individual level and for variables at the FU level. Generally, a higher level is adopted in the univariate analysis, allowing the variables to enter the multiple analysis. In the multiple modeling, a significance level of 5% was adopted. The text has been revised to make this point clear.*

8. 262: I don't understand the meaning of "with fixed coefficients (varying between FUs)" here. I think the FU-level random intercepts and random coefficients are the only parameters that vary between FUs, no?

*Thank you for this comment. It was a mistake: “(varying between FUs)” was excluded.*

9. 266: What was the acceptance threshold for comparisons based on AIC? Also, based on the described methods, I'm not clear on which comparisons would involve non-nested models. Is this only for the random coefficients?

*There is no threshold stablished for comparisons based on AIC. This approach is used to determine the influence of a variable introduced in a model when the smaller model is not a subset of the model with more variables. The model with the lowest number of AIC is in general assumed as the best, because it includes the likelihood log estimation and a compensatory term for the number of variables, resulting in a more parsimonious model. On the other hand, for nested models, that means, when the larger model (with more variables) contains all the variables of the smaller model, the likelihood ratio test is recommended. The text has been revised to make this point clear.*

10. 119: Isn't the offset the number of confirmed cases *with known clinical outcome*, rather than the total number of confirmed cases per se? On this point, given that only 79% of confirmed cases have known clinical outcome (line 287), I think a very brief discussion of possible biases in missing data would be appropriate (probably in the Discussion section). E.g. Is it possible the ~20% of cases with unknown outcomes are biased toward survivors, or exhibit temporal trends in fatality different from the 79% with known outcomes?

*Thank you for the opportunity to discuss this point. Despite the loss of 21% of the cases of VL in the period due to lack of available information on the clinical outcome, considering that, the registration of the diagnosis of VL in the death declaration triggers mandatory investigation by the epidemiological surveillance system in Brazil, we believe that the number of fatal VL cases losses was negligible and that these results are not likely to be subject to selection bias. In addition, 32% of the cases with unknown clinical outcome were cases transferred to another municipality, that means that they were reported in duplicate, that is, they have were included in the database. This* *reflection was included in the discussion section (pages 282-289).*

11. 195: It still seems unnecessary to use a GAM here. The temporal-trend results later presented are based on a GLM (Table 2, line 303), and the temporal term in the univariate and multivariate analysis is likewise a linear term. Why not just use the linear term (GLM) for all temporal analyses?

*In this case, adjusting the time using GAM or GLM are analogous approaches, given the perfectly linear relationship. As previously presented, the GAM model was used only to observe the shape of the relationships between the variables and the outcome, as an analysis prior to deciding how to analyze the variables in the linear models (whether dichotomized, truncated or not).*

12. 215, 234: I think "expected VL cases" should instead be "observed VL cases"? "Expected" implies a prediction or a model-estimate, but I think the offset is just the observed number of cases per FU.

*Thank you for the opportunity to clarify this point. In this case, the term "expected" was used to inform the direct correction made to the data in order to adjust the age distribution to the standard population (used as the control of the age distribution). The recommended term is “expected number of cases”, that means, the number of expected cases assuming an age distribution, in this population, equal to that observed in the standard population. To avoid this misunderstanding, we changed the term to "the calculated number of cases if age distribution were equal to the standard population".*

13. 243: "greatest association" based on what metric?

*Thank you for the opportunity to clarify this point. “Greater association” in terms of a lower p-value in the univariate model (higher significance). It was clarified in the text.*

14. 259: Related to point #1 above, it's not clear to me why the discretization should aim to split a variable into segments where the relationship with the response is linear, per se. Within-factor linearity is not a model assumption. As noted above, I suggest omitting the discretization step, but if retained, this particular approach should be better justified/explained.

*Unfortunately, at this point we have to disagree. The models* *that we opted to use to estimate the average effects assume linearity between the explanatory variables and the outcome, as indicated in their designations: “Generalized LINEAR model” and “Mixed LINEAR model”. Relationship between explanatory variables and outcome was not evenly linear for all variables in this study, as informed by GAM models. Thus, categorizations were used exactly to guarantee respect to linearity assumption. If linearity was detected by GAM, the variable was explored in its original form. In this sense we would like to point out that our interest is not only to adjust the variables to the outcome, which the GAM model would do in the best way, but to obtain average estimates that help us to explain the outcome with reasonable interpretability.*

15. 285-286: Clarify the level that the average/range are taken across (e.g. years, FU, or both).
*This information was added: “The national average VL incidence in the 11 year-period”.*

16. 304: The 95%CI uses a comma decimal separator whereas the rest of the text primarily uses a period (though there are also comma separators for incidence and case fatality in Table 1).

*The decimal and thousand separators have been standardized. We appreciate the observation.*

17. 318: Clarify "has a low probability of occurrence in its a posteriori distribution".

*In this context “a low probability of occurrence in its a posteriori distribution” means “little mass of data under the* curve” or “*small amount of data under the curve” (it was clarified in the text).*

18. 335: The subheading "Individual level analysis" in the Results section corresponds to subheading "Multilevel analysis" in the Methods section. Suggest using a common subheading. "Multilevel analysis" makes more sense.

*Thank you for this observation, the subheading was changed.*

19. 337: Not clear what the significance of "an age up to 14 years" is here. Could just omit and report only the median/IQR (16 years, IQR 2-39).

*The suggestion was accepted.*

20. 338: A 75% IQ is unconventional. Is this instead the 50% inter-quartile range, i.e. 25-75%?

*Exactly. The interquartile range (****IQ****) = middle 50% = the difference between 75th and 25th percentiles. We changed the* [*interquartile range abbreviation*](https://www.google.com/search?client=firefox-b-e&sxsrf=ALeKk029Elh0i45cgaisMcs0HEFNypRZqg:1616848986800&q=interquartile+range+abbreviation&spell=1&sa=X&ved=2ahUKEwj-xo3sv9DvAhVuILkGHVcxA7AQkeECKAB6BAgBEDU) *to IQR.*

21. 341: Consider changing "totalized" to "totalled".

*The word was changed.*

22. 347: The reported median age of the cured group (13 years) does not match the 95% CI (25-75 years).

*Many thanks for this observation. It was a typing error. The correct is
13 years (95% CI 2-36 years).*

23. 345-348: The reported medians and 95% CIs in onset to treatment delay in the cured (33, 16-76 days) and fatal groups (30, 15-59 days) are almost certainly *not* consistent with a significant difference between the groups. Perhaps the 95% CI are actually IQR?

*Many thanks for this observation. You are absolutely right; the range is the 25-75% interquartile range. The manuscript was corrected.*

24. 350: Add full test results anytime a p-value or claim of significance is made. Generally this includes test static, degrees of freedom, and p-value. Could also note the test type if it's not clear from the Methods. Also, should use e.g. p < 0.001 rather than p = 0.00.

*We accept the reviewer suggestion and our option was to present the full description of the tests and significance adopted in the methods section.*

25. 361: Clarify the correlation metric used here (presumably R^2).

*The correlation coefficient used was Spearman. The information was added to the text.*

26. 384: This odds ratio (6.4) seems surprising given the data in Table 2 (I calculate a raw odds ratio of 1.13; CFRs: 8% vs. 13%). Of course, the ORs in Table 2 are based on a model with year and FU-intercepts, so could be different. Just worth double checking.

*We have double checked and confirmed the odds presented. Note that, even after adjusting for other variables, this effect dropped slightly, but the effect is still high (Table 3). As you have mentioned, it is important to note that, in addition to age and UF's random effect adjustment, the models adjust the number of VL cases, considering the “expected” cases (adjusted by age distribution of the standard population) in each FU.*

27. 388-390: I'm not clear on why these 3 variables (race, locale, case classification) aren't presented in Table 2. Perhaps because they are statistically non-significant? Would be preferable to present results consistently regardless of significance level.

*The variables were included in Table 2.*

28. 399: Should say "Fig 5l" instead of "Table 5l"

*Many thanks for this observation. The manuscript was corrected.*

29. 411-412: The text and Table 2 imply that CFR declines with more medical doctors per capita, but Fig 5i suggests it generally increases.

*Thank you for discussing this point. We do not see the results mentioned as antagonistic. Figure 5 and Table 2 illustrate different analysis involving the number of doctors. In Figure 5i, “number of doctors / 1000 inhabitants (in each FU)” is a continuous variable. The figure shows an increase in lethality with the increase in the number of doctors up to 1.5 doctors / 1000 inhabitants, after this value, the direction’s effect changes toward to a slight decreasing tendency on the response, although the confidence interval is quite wide. Because of this break in linearity, we chose to categorize the curve at this inflection point. In this way, the model estimates no longer the influence of the increase of one unit on the number of doctors, but the effect of “having more than 1.5 doctors / 1000 inhabitants” or “having less than 1.5 doctors per inhabitants”, and this relationship is inverse with the outcome (VL case fatality), the more doctor per inhabitant, the lower the lethality, as signaled by the downward curve after 1.5 (Figure 5i).*

30. 437: The use of "multiple hierarchical analysis" in the caption for Table 2 is confusing given that the models are described as univariate. Could change to something like "generalized linear mixed models with FU-level random intercepts".

*The suggestion was accepted.*

31. Table 2: I don't understand the meaning of "VL cases (%) according to..." in some of the variable labels. I think can just be omitted?

*The suggestion was accepted.* *This information was added to make clear that the values presented in the columns refer to the absolute number and percentage of VL cases stratified according to each variable, but we agree that this can be understood.*

32. Fig 1. The use of the two scales (deaths vs. CFR) but single axis in the left panel is awkward. And the right panel should be transformed to the scale of the response (i.e. probability or percentage) and given an informative y-axis label instead of "spline(year)". Also, isn't the right panel showing a temporal effect on VL case-fatality instead of on "VL Deaths" per se (given that the model uses cases as an offset term)? I think it would be preferable to show the death numbers in one panel and the CFR raw data (red line) and model predictions (spline panel but on the response scale) together in a separate panel.

*The bars with the absolute number of cases (left panel) were excluded. We emphasize that the main information to be extracted from this graph is the form of the relationship (whether linear or not), the size of the effect was measured using a linear model, as informed. The y-axis label was informed.*

33. Fig 2. The breaks for the colour scale in the right panel seem to be truncated too low (15%) — only about halfway up the scale.

*The figure was edited.*

34. Fig 3. Add axis scales and labels for panels (a) and (b). Also, just an aesthetic point, try to make panel (d) the same size as (c).

*The figure was edited.*

35. Fig 5. Consider standardizing the range of the y-axis so that it's possible to visually compare the relative magnitude of the effect size across panels/variables. Also, the y-axes should be transformed to a meaningful scale (i.e. CFR, probability or percentage). The current label "spline" is not informative. Also, not clear why the x-axis data is depicted as dashes along the bottom in some panels but not others. Regardless, I'd strongly suggest adding the actual data points (x and y) instead to every panel so readers can access the fit of the splines.

*The standardization of the y-axis (the same scale) would impair the visualization of some relations, considering the differences among variables. As mentioned previously, the information to be extracted here is the form of the association as the effects were later estimated in the linear models.*

36. Fig 5c. The panel depicts a split-point at 1400 days (also noted line 391), but Table 2 suggests this variable was treated as a continuous linear predictor.

*The GAM model did not show significance for values above 1400 days, note that values for this segment belong to the confidence interval that contains the zero value. Thus, this variable was truncated at the point of 1400 days and values above this point were discarded. After this first approach, the variable was analyzed assuming linear relationship with outcome. It was clarified in the manuscript.*

37. Fig 6. What is nm_uf? Wasn't this a parameter of the spatial model? Also, suggest consistent use of subheadings — e.g. why use a subheading (Basic Model) for panel (a) but no subheading in (b)? Also, add label (including units) to the x-axis.

*The figure was edited and the subheadings were standardized.*

38. The biggest remaining issue is the presentation/interpretation of results. As far as I can tell, none of the FU-level variables were selected into the final multilevel model. The 7 variables that were selected were all individual-level factors (age, symptoms, gender, etc.) (Table 3). To me the simplest interpretation of this result is that, after accounting for individual-level factors, *none* of the FU-level variables (# hospital beds, garbage collection, proportion children, etc.) account for a substantial proportion of variation in case fatality. The authors do note this caveat in the Discussion (633-636). "These results suggest that the geopolitical structure represented by the FU are insufficient to reflect the differences within the unit, since the magnitude of the effects of contextual variables were generally weak, losing their importance in multiple models." but nonetheless suggest that some of the FU-level variables are important predictors of case fatality in other parts of the manuscript, e.g. "...unavailability of emergency beds and health professionals (the last two only in univariate analysis) were identified as related to VL death risk." (line 43); "Lower VL incidence was also associated to VL case-fatality, suggesting that unfamiliarity with the disease may delay appropriate medical management" (line 45); "The indicator of social and economic development, access to garbage collection, was confirmed as protective against the risk of dying from VL." (line 603); "higher rates of elderly people and life expectancy, both related to a greater number of elderly people in population, were directly related to the risk of dying from VL" (line 610). I think these conclusions are overstated given the analyses presented. It could of course be the case that some of the FU-level variables affect CFR indirectly through their impact on individual-level factors. E.g. Perhaps some of the health-related FU variables (e.g. doctors per capita) indirectly affect CFR through their effect on onset-to-treatment delays. But this sort of mediating relationship could/should be tested directly. Along the above lines, particularly with respect to FU-level variables, I think a more focused analysis would be preferable. Potential causal pathways can be identified from the outset, and the relevant FU-level variables chosen in a more targeted fashion. In the current analysis, I think there's not much to be learned from the inclusion of FU-level "Gender proportion" in the same model as individual-level patient gender, or FU-level "Proportion under 5 years" in the same model as individual-level patient

*Initially, we would like to state that the variables at FU level were selected from the multilevel model, however, as noted by the reviewer, they did not remain in the best fit model (they lost significance). Observe that those at the individual level, as well as the random effect at FU level, capture more of the outcome variability than the contextual variables (at FU level)l. However, it is important to recognize that this observation does not necessarily mean absence of influence of the contextual variables, but the presence of a lesser degree of influence on outcome. Another point that needs to be observed is the limit of each study according to its design, an ecological study, like this, is not able to establish a causal relationship between explanatory factors and outcome. In this sense, we have only an indication, in average terms, about the associations between some variables at the individual and FU levels and outcome. An essential point to be highlighted is the existence of a lot of variability into the FU level, related to vulnerabilities, disease risk, and access to the health system. Thus, the variables measured at this level of aggregation (FU) did not allow us to detail these associations. As we have shown, there is indeed an important part of this variability within each FU explaining the VL case fatality, however, the variables used in this study do not seem to be the most involved with the differences observed in the outcome. An analysis using a smaller scale unit (municipalities or basic health units, for example) would be ideal, however, there is no repository available gathering these data. We believe that the studies with different designs complement each other, providing inputs about the influence of contextual and individual sources. The contribution of this study is to highlight that the determinants of VL case fatality in Brazil differ depending on the spatial scale of analysis, which is important to support local control and prevention actions in public policies. Finally, about the inclusion of variables apparently equivalent, but measured on different levels, into the same model: we understand that they address different influences. For example: age has a specific influence on the health-disease process, at the individual level, however the proportion of children under 5 years in a region may be related to contextual factors, such as the vulnerabilities of such region. In this example, regions with a younger age distribution tend to be less socioeconomically developed. In other example, in the comparison between two VL subgroups with the same age, sex, symptoms, etc., but living in two regions with different conditions of access to the health system and different social-economic status, we find a higher case-fatality rate in the less developed region.*

39. Related to the methodological concerns about unnecessary discretization, some of the conclusions regarding FU-level variables (also noted in point #1 above) are not evident when looking at the splines in Fig. 5. E.g. The authors conclude "Lower VL incidence was also associated to VL case-fatality, suggesting that unfamiliarity with the disease may delay appropriate medical management" (Abstract, line 45), but the spline relationship in Fig 5d does not suggest a simple unidirectional relationship between incidence and CFR. The spline relationships between CFR and emergency beds, number of family health programs, and garbage collection are similarly complex.
The most important step to clarify these relationship would be to show the underlying data in the spline plots. Assuming the splines are a faithful representation of those data, the conclusions should be revised to add nuance, and focus on describing the actual relationships, even if they are non-linear and complex, and de-emphasizing the discretized results.

*As we have already presented previously, the approach based on the initial assessment of the presence of linearity by GAM models is well established in the literature (*[*https://m-clark.github.io/generalized-additive-models/appendix.html#references*](https://m-clark.github.io/generalized-additive-models/appendix.html#references)*;* [*https://rss.onlinelibrary.wiley.com/doi/full/10.1111/j.1467-9876.2005.00510.x*](https://rss.onlinelibrary.wiley.com/doi/full/10.1111/j.1467-9876.2005.00510.x)*)* *and was essential to guide the handling of the variable without violating the assumption of the models (linearity between explanatory variable and outcome of interest).*

*The reviewer is correct, most of the relationships are complex. Considering it, all of them were analyzed based on the association shape and reported in detail, as following (lines 387-430): “Considering a non-linear association between standardized VL incidence and VL case-fatality (Fig 5d),* *this variable was categorized as following: up to 60, 60 to 160, and above 160 cases per 100000 inhabitants. A significant difference was observed in proportion of FU of origin with more than 160 compared to less than 60 VL cases/100000 inhabitants between groups, higher among cured VL cases in comparison to VL cases with fatal outcome.” “…the number of emergency beds/100000 inhabitants was significantly associated to VL case-fatality up to 1000 beds/100000 inhabitants (Fig 5e). The effect was estimated up to this setpoint (Table 2) and an inverse association was confirmed: the greater the number of emergency beds, the lower the VL case-fatality (Fig 5f). Inversely, the number of hospital beds per 100000 inhabitants exhibited a linear association with VL case-fatality. The number of multi-professional teams working at Family Health Program (FHP) was inversely related to the risk of dying from VL, for the interval of 200 to 600 FHP teams/FU. Thus, this variable was evaluated up to the limit of 600 FHP teams/FU, dichotomized at <= 200 and> 200 FHP teams/FU. The same occurred with the number of medical doctors, a progressive effect up to 1.5 doctors per 1000 inhabitants, stabilized afterwards (Fig 6i). This variable has been dichotomized and a protective effect against VL case-fatality was confirmed for more than 1.5 medical doctors/1000 inhabitants. Health expenditure higher than 700 reais per capita/year, around 134 dollars, was inversely related to VL case-fatality (Fig 5j), the setpoint used for this variable dichotomization. However, using this setpoint, a significant association could not be confirmed in univariate analysis (Table 2). Concerning to garbage collection, a linear association with VL case-fatality was observed for rates higher than 86% (Figure 5h). As shown in Table 2, for rates above 93% of garbage collection, as the access to the service increases, the VL case-fatality decreases.”*

*Based on a thorough and individualized analysis that took into account the assumptions required for the establishment of associations, we are confident that the splines faithfully represent the data. In addition, there is evidence in the literature to support that the direction of effects are maintained when using continuous or discretized variables, as long as the categories are defined based on strong basis. In this sense, we believe that the GAM models are quite useful in supporting these cutoff points. Attending to the reviewer request, we have revised the description of all relationships between contextual variables and VL case-fatality (lines 391-439) and these associations with outcome were presented as possible and generating hypotheses and not as established, along the discussion and conclusion sections.*
