# Supplementary material for: Inequalities of visceral leishmaniasis case-fatality in Brazil: A multilevel modeling considering space, time, individual and contextual factors
Source: PLoS Negl Trop Dis. 2021 Jul 1;15(7):e0009567. doi: 10.1371/journal.pntd.0009567 (PMC8279375; doi:10.1371/journal.pntd.0009567)
Supplement: Attachment — Submitted filename: answer to reviewers_thirdReview.docx [file pntd.0009567.s006.docx]

May 20, 2021

Alberto Novaes Ramos Jr, M.D., M.P.H., Ph.D.
Guest Editor
PLOS Neglected Tropical Diseases

**Manuscript: PNTD-D-20-01885**
*"Inequalities of visceral leishmaniasis case-fatality in Brazil: a multilevel modeling considering space, time, individual and contextual factors"*

Dear Dr. Alberto Novaes

We are grateful for the thorough analysis that reviewer 3 has carried out on this study. At this point, we believed that our choices for this methodological approach have been sufficiently presented. All these choices can be criticized, which is highly desirable, considering the main purpose of a scientific dissemination, that is to contribute to the understanding of reality from multiple views. However, it is important to emphasize that these are justified choices and not methodological inaccuracies. In this sense, we confirm our intention to have this approach published here or in other journal, and discussed with the scientific community as a whole, and not just with the reviewer 3, who has already expressed his preference for other approaches.

We thank you for considering this study for publication and all inputs provided.

Sincerely,

Gláucia Cota.

**Answer Letter to Reviewer 3**

1.In my opinion there remains significant problems with the clarity of the methods, and with the methodological approach of discretizing or truncating explanatory variables based on visual assessment of GAM models. Discretizing/truncation based on GAM: The most problematic use of discretization/truncation is for the variable Emergency beds, where, as far as I understand, the authors decide based on the GAM model to exclude data above a setpoint of 1000 emergency beds / 100k inhabitants. The resulting negative relationship between emergency beds and fatality (which is repeatedly referenced, e.g. lines 43, 70, 410, 582, 636), is in my view completely meaningless because the authors have specifically chosen only to analyze a segment of the curve in which the trend is negative, while ignoring the next segment in which fatality generally *increases* with the number of emergency beds. This problem of conclusions influenced by arbitrary truncation also applies to the variable "Number of FHP Teams". More generally, it's still not sufficiently clear how the authors have chosen the split points, or how they decide between discretization (e.g. Age) and truncation (e.g. Emergency beds).

*Again, we strongly reaffirm that it was not arbitrary truncations but based on the interpretation of the GAM models. Discretization of continuous variables is an established strategy to facilitate the understanding of the influence of a given variable on outcome. The choice of cutoff points based on statistical models is, in this sense, the most correct alternative. In the GAM model, these points are identified by the change in direction of the effect of a predictive variable on the outcome.*

*In relation to truncation, choosing a range of values is the recommended strategy for reducing type II error. GAM is able to demonstrate very clearly for which predictor values there is a significant effect on the outcome. In addition, to confirm the correctness of this approach, we tested the models with different cutoff points, and with the variables in their continuous format. From the point of view of estimates and statistical significance, the difference was very small, which reinforces that the interpretation we propose of the effects is correct. In addition, discretization at inflection points prevents the violation of the assumption of linearity between the predictor variable and outcome, as already pointed out by us.*

*In turn, the negative relationship between the number of beds and the outcome has nothing to do with the discretization carried out. This is related to possible confounding or interaction effects not considered in the study. Figure 5e clearly shows that there is no effect of the variable above 1000 (confidence interval contains zero), in addition, the confidence interval is quite wide, expressing the inaccuracy of the estimates for values above 1000 beds per 100,000 inhabitants. Therefore, the truncation of this variable was not arbitrary, but based on evidence from the GAM modeling.*

2.The assessment seems to somehow depend on whether the GAM confidence interval crosses zero, e.g. "... inferring in which ranges of values this association is significant, which can be declared when ranges of values of the curve do not belong to the estimated confidence interval and contain included zero value." (259-261). But it's not clear to me how to get from this description to the specific split points in Fig 5. E.g. For variable Standardized VL Incidence, there are split points at the 2 inflection points on the curve, which seem unrelated to the CI crossing zero. In contrast, for Age there is a split point where the CI crosses zero and *not* at the clear inflection point around age 10. For Family Health Programs, the first split point is at an inflection point and the second occurs where the CI crosses zero. In the response to the previous review (point #1) the authors suggest "There are countless ways to apply the GAM models for this purpose", and go on to explain several seemingly different approaches, but this did not help me understand which approach was actually used for which variables, or why.

*The ranges of values in X-axis with a linear relationship to the outcome and the ranges where the effect is null are two different observations taken from the graphic model GAMM. As textually informed in the manuscript, the cutoff points in X-axis were defined based on the points of change in the direction of the effect (points of inflection of the curve) and changes in the response effect (curve crosses the horizontal line of zero value). As an additional information, if the confidence interval of values estimated by the curve contains the null value, the effect on the response will be null for this range of values in X-axis. In the VL incidence example (Figure 5d), there are two points of inflection of the curve that define three distinct ranges of values. Observe that all incidence values could be included in analysis, compared as three different blocks, as a linear relationship with the outcome was observed for all the three intervals. It can also be observed that in the end of the curve, around 60 and over 250 cases / 100,000 inhabitants, the effect of the incidence is zero on VL case fatality. However, this lack of effect on these specific points does not invalidate the presence of effect on other sections of the curve, on the contrary, it identifies the point where there is a complete change in the direction of the association. In turn, for age (Figure 5b), there are two ranges of values clearly identified by the different slopes of the curve, and the value “20 years” is the exact point of change in the slope direction. Again, this point was chosen because it marks the change of direction on the slope. The fact that exactly at the “20 years-point” the effect is null only confirms that at this point there is a complete change in the direction of the trend and not only in the degree of the association. For the variable “Number of teams of health family program” (Figure 5f), the truncation after the value of 600 teams was decided because the zero effect (red line) is within the confidence interval of the entire curve after this point.*

3.I also think the interpretation that regions of the GAM confidence interval that cross zero indicate "a null effect in that region" (e.g. 259-261, 386-387) isn't quite correct, nor is it meaningful to visually assess regions of a GAM curve where the relationship is "significant". The GAM fit lines are showing the predicted mean value of y (+/- confidence interval) on the transformed scale (e.g. logit) at a given value of x, relative to the overall mean value of y. They're not showing the *effect* of x on y (i.e. the slope of the relationship) at a given value of x (though this can be interpreted from the slope of the fit line). E.g. for variable Medical doctors (Fig. 5i), the GAM confidence interval overlaps with zero in the region of about 0.95-1.2 doctors per 100k inhabitants, but the slope of the relationship between Medical doctors and fatality is still clearly positive (and relatively linear) in this region, so I don't see why/how we would use the fact of an overlapping CI to inform discretization.

*Unfortunately, we have to disagree with the reviewer regarding the interpretation of the GAM graphs. The plots generated are partial effect plots: they show the component effect of each of the smooth or linear terms in the model, which add up to the overall prediction. They also show the 95% confidence interval for the mean shape of the effect. As confirmed by the reviewer, the curve and respective intervals represent the estimated effect of a non-linear function of variable x on the outcome. The graph shows the original scale on the Y axis, therefore, the estimate of beta (β) related to f (x), on the risk of a case of VL result in death. Thus, if Beta is null, for a given value of x, there is no effect of x on the outcome. That means that if the beta estimate is zero (it belongs to a range that contains zero) for some values of x, x has no effect on the outcome in this range of values.* [*https://noamross.github.io/gams-in-r-course/chapter2*](about:blank)*. In figure 5i, the point chosen was that one where there is a change in the curve slope. In this case: up to 1500 medical doctors/1000 inhab there was an increasing relationship with the outcome, and after that point, the relationship becomes stable.*

4. Similarly, as I noted in my previous review, "it's not clear to me why the discretization should aim to split a variable into segments where the relationship with the response is linear, per se." I understand that GLM's have a linearity assumption, but if an explanatory variable is discretized prior to analysis there is not an assumption of linearity *within each discrete category* of that *variable per se.
Precisely, it is exactly the prior analysis by GAMM using the original continuous variable that allowed us to identify those in which the assumption of linearity was violated. In turn, the discretization of a variable with a linear association with outcome did not change the nature of association compared to the analysis using the original continuous variable. In the present case, it only assisted in understanding how the association occurs.*

5. I can see the logic of using GAM models to identify linear segments if the purpose is truncation — to exclude values outside the linear segment (though I think think truncation is a bad approach generally). But I don't see the logic to using GAM to identify linear segments if the purpose is discretization. E.g. the variable "Interval between symptom onset and reporting" was dichotomized using a cutpoint of 200 days, which generates two roughly linear segments based on the spline. But an analysis that uses this dichotomized explanatory variable doesn't require that each segment is linear... the analysis only sees two groups, >=200 or <200, with no relation to the continuous variable "Interval between symptom onset and reporting". So why choose split points specifically based on linearity here?

*In this case, it seems to be a misunderstanding involving linearity and lack of effect of the variable on the outcome. Truncation was done exclusively at points that separate significant and non-significant effects, thus, part of the predictor variable that does not help to explain the outcome was excluded from the analysis. When there were points of inflection (growth / decrease) or change in the relationship direction (positive / negative), we have chosen to categorize the variable in order to facilitate interpretability and to overcome the violation of non-linearity. In the case of the variable "Interval between the onset of symptoms and reporting", there are clearly different effects, increasing up to 200 days and stability afterwards.*

6. I'm sympathetic to the authors' previous response (point #1) about using discretization to aid in interpretability, but this still requires more nuance and a clearer explanation of the methods than are presented here. Finally, in response to my previous comment about discretization, the authors respond (#39) "... the approach based on the initial assessment of the presence of linearity by GAM models is well established in the literature...", citing the reference section of a book "Generalized Additive Models" and a paper "Generalized additive models for location, scale and shape". I looked through the book and the paper the authors cite, and see no relation to the particular discretization approach used here. I haven't checked every single reference in the book, but the authors can clarify the relevant references if necessary.

*In terms of estimates and statistical significance, the discretization approach does not alter the conclusion about the nature of the association between a given variable and outcome. In the case of the analyzes presented in this study, the choice fulfilled the objective of meeting the assumptions for the use of the models. The reference cited is just one example of the multiple possible approaches to perform discretization and the use of GAMM for this purpose.*

Other things to clarify:

7. Line 244: Clarify which p-value is being referred to in the line "those with the lowest p value were selected for the multiple models". Is this the p-value from the univariate models described below (i.e. also adjusting for time, FU, and age distribution), or the "parametric and non-parametric tests" described immediately above (237-243)? The authors' response letter (#13) indicates it's the univariate models but this is not clear in the text itself.

*Thank you for this comment. The information was added in the manuscript.*

8. Should explicitly state within the manuscript or appendix, probably in a table, which variables were excluded on the basis of multicollinearity. And clarify whether these variables were also excluded from univariate analysis. I'm assuming not, since e.g. child mortality and life expectancy are both included in the univariate results Table 2 (Results) despite looking nearly perfectly correlated (Fig 4).
*As informed in the manuscript, in case of multicollinearity identified, the variable with the highest p-value in the univariate modeling was excluded exclusively from the multiple analysis, being maintained in the univariate analysis. Our option in this case was to show the correlations through a diagram (Figure 4), where the correlations range from –0.80 to -1 and from 0.80 to 1.*

9. Line 257: The GAM(M) models used as part of the multilevel analyses should be more fully described here, e.g. Error distribution (presumably binomial) *In a generalized model, the distribution of the Error is not pre-defined, but the distribution of the response variable, which in this case is Binomial. The information was included in the manuscript.*

10. What type of spline (e.g. thin plate, cubic, etc.). E.g. The default in the spline function s() in the mgcv package is "thin plate regression spline". *The default function of the s () function of the MGCV package was used: thin plate regression spline. The information was included in the manuscript.*

11. Is a time term included as in the GLMM models? Are there random effects included (e.g. FU)? If so how are these specified, as there are multiple possible approaches with GAMMs. The Results section notes both GAM (385) and GAMM (392), so it's unclear where these are mixed models or not.

*As informed in lines 249-252, the variable “reporting year” (time) and a random term at FU level were included in the models as they were confirmed as related to outcome in the previous spatial and temporal analysis. There are two acronyms properly defined in the text: “GAM model”, generalized additive mixed modeling - used for temporal analysis and “GAMM”, generalized additive mixed modeling, used in all other analyzes.*

12. Line 268: There are various approaches to stepwise variable selection. This sounds like "forward variable selection", but should clarify explicitly here. I.e. I'm not positive from the description that it's not a bi-direction selection approach, where variables could be added or removed at each step.
*The initial procedure was forward manual variables selection, however we tested the output and input of the other variables at each step until the entry of the least significant variable in the univariate analysis. The information was added to the manuscript.*

13. Line 269: Relating to the point about explicitly stating which variables were omitted on the basis of multicollinearity, the line "variables were introduced one by one into the basic model in decreasing order of significance, **considering the multicollinearity and the particularities detected in the previous analyzes**" is not sufficiently clear to be reproducible. It's difficult to judge the exact correlation values from Figure 4, but for example, MedicalDoctors looks potentially highly correlated (|r| > 0.85) with lots of variables: Sewage, LifeExpectancy, Water, Garbage, Urbanization, ChildMortality, and Children<5. Is the exclusion of correlated variables done at the very beginning, such that 7 of these 8 variables would be immediately excluded from all further analyses (all except the one most strongly related to fatality based on the univariate tests)? Or are the exclusions updated at every step such that, e.g., the 7 variables correlated with MedicalDoctors are only excluded after a step in which MedicalDoctors is retained in the multiple model (if any)?
*As informed in the manuscript, the significant variables were introduced one by one into the basic model in decreasing order of significance.* *The exclusions were made pair by pair, in case of two related variables are together in the same model. All excluded variables were retested, one by one, in the final model.*

14. Line 273: I'm still not clear which comparisons would involve non-nested models. If a single variable is added at each step, shouldn't the original model always be a subset of the new model (i.e. the new model only has one extra variable)? Consider adding an Appendix to more clearly illustrate the variable selection approach.

*We have double checked the analyzes performed and we have confirmed that in this study the contribution of all added variable was assessed by testing the likelihood ratio between nested models. We have excluded the mention to the analysis by non-nested modeling, a methodological approach proposed during the study planning and before the study execution.*

15. In my opinion there remains significant problems with the presentation of results, and ambiguity as to why results for some variables/analyses are not presented.
Table 2 (Results):

I still don't understand why some variables presented in Tables 1 and 2 (Methods) are missing from Table 2 (Results) (likewise for Fig. 5, for the continuous variables), e.g.:

- interval from treatment to death, race proportion, illiteracy, population growth rate, average household income, sewage, PIB, HDI, health units (result described line 407), emergency beds (result described line 408)
*Variables highly related to others of the same group (demographic or health care access) were not shown in the table, only cited in the text with the information that no association with the outcome has been identified. For example, the percentage of illiterates is including in the variable “schooling”, race proportion is related to variable “race”. An explanation was included in the text.*

16. Table 2 (Results): For categorical variables, in the "Univariate analysis" column there should only be odds ratios in cells *other* than the (base) level. This is currently the case for variables with 3 categories (e.g. Number of VL symptoms (%), Standardized VL incidence), where the odds ratio for the base level is replaced with "-". But for all the variables with 2 categories (Age, Gender, etc.) the odds ratio is placed on the line corresponding to the level indicated as "base", which makes interpretation ambiguous. E.g. For "Gender" the odds ratio is placed in the line corresponding to "Female (base)". Does this odds ratio represent the risk for females relative to males (as indicated by the placement of the OR), or males relative to females (as indicated by the designation "Female (base)")? Suggest consistent use of "-" for the base level cells for clarity.

*Table 2 was reviewed according to the reviewer request.*

17. Lines 351-360: Why present these results from the "initial global analysis" only for these 3 specific variables (age, onset to symptom delay, diagnostic criteria), and not all the other variables? It would be preferable to present all results in the same way. But at a minimum, if you choose to present only certain results for a given type of analysis, it should be clear to the reader how specifically you chose which results to present and which to omit. More broadly, I'd suggest omitting these 'global analysis' results altogether, as they're describing analyses that are basically just the inverse of the univariate models (i.e. they use patient outcome as an explanatory variable and the demographic/diagnostic variables as response).
*Thank you for the opportunity to clarify this point. Variables chosen to be described nominally are the main clinical characteristics of patients diagnosed with VL. The reason is to characterize the groups under aspects easily identifiable by the health professional. It was necessary because in the Table 2 several variables are presented as categories, which took the place of the central values (median or mean), an information with which professionals will have greater identification.*

*.*
18. Line 358: These categories of diagnostic confirmation (parasitological test, IFAT serological testing, other serological testing) are missing from Table 1 (Methods)

*These variables were included in Table 1.*

19. Line 365: Clarify the line "high correlation among several contextual variables (Figure 4), such as degree of urbanization and proportion of the population up to 5 years old, correlated to many other variables." Based on Fig 4, the variables Urbanization and Children<5 don't appear to be correlated above 85%.
*We have changed the example of correlated variables in the text.*

20. Line 367: Clarify whether this also accounts for negative correlations (e.g exclude |r| > 0.85) ?
*Yes, the modulus | r | means that r is> -0.85 or> 0.85.*

21. Lines 385, 387: Should be referencing Figure 5 instead of 6 here I think

*Thank you for this correction, the figure number was corrected.*

22. Line 394: The term "crude effect" is a bit confusing here, because the odds ratios in Table 2 are from univariate models that also include terms for FU and time, and adjust for age structure. They're not really "crude". Though based on the counts in Table 2, the crude effect for age (1911/12600)/(820/16506) = 3.05 is almost the same as the univariate odds ratio (3.01).
*To solve this misunderstanding, we have included a term definition: “crude effect” was used to refer to the effect of only one variable in addition to the temporal term and random effect already included in the model.*

23. Line 399: change "discharge" to "discarded"	*The word was changed.*

24. Lines 402-404: Rephrase this result to clarify. If I understand correctly, this result is treating VL incidence as the response variable and patient outcome as the explanatory (like the "initial global analysis" on lines 351-360)? Suggest just presenting the univariate results here for consistency and clarity.

*This statement refers to the univariate analysis presented in Table 2. The sentence was rewritten.*

25. Line 411: I think this ref should be to Fig 5e (Emergency beds) instead. There are also references to Fig 6 that should be to Fig 5 (e.g. line 418). *That’s correct. The corrections were made.*

26. Figs. 1b, 5: I don't think "Effect" is a sufficient y-axis label here. Assuming these plots come from e.g. the plot() function in R used on a MGCV gam model object, the y-axis shows the predicted values of the y variable on the transformed scale (e.g. logit in this case), centered to a mean of zero based on the model intercept. A more accurate label would be something like "logit (Case fatality), centered".
*As mentioned earlier, the graph represents the partial effects on the outcome on the original beta and non-exp (beta) scale. The Y axis is not VL case fatality, but the effect on VL case fatality, so we can assess the significance of the explanatory variable plotted on the outcome, in ranges of values whose curve does not belong to the confidence interval that contains the zero value between its possible values.*

27. The latest revisions to add nuance to interpretations of results that differ between univariate and multiple analyses are a step in the right direction, but in my view there remain conclusions that are not justified by the methods/results, and a lot more nuance is needed for certain analyses. The biggest problem relates to over-interpretation of analyses of variables that were truncated arbitrarily in my opinion) prior to univariate analysis, particularly "# of Emergency beds" and "# of FHP units" (e.g. 580-584, 635-638). E.g. The authors conclude: "The link between access to diagnosis and health facilities and VL case-fatality is also suggested by the inverse association found in univariate analysis between availability of emergency beds, FHP teams and medical doctors and death from VL, which suggests that the robustness of the health system is directly related to the VL clinical outcome." (580-584) and "The variability in the installed structure of the public health system, expressed by the number of emergency beds and health multi-professional teams, in addition to the ability of the health system to recognize the disease, which is directly related to the regional incidence of VL, appear as additional markers of the risk of death." (635-638) For "Emergency beds" and "FHP Units", the splines (Figs 5e, 5f) indicate an inverse relationship only in certain regions of the curve, and the authors have simply excluded later regions where fatality appears to increase with the variable of interest. This is not at all rigorous in my opinion. The conclusion regarding the relationship between VL Incidence and fatality ("Lower VL incidence was also associated to VL case fatality, suggesting that unfamiliarity with the disease may delay appropriate medical management" (45-47)) should also be more nuanced given the relationship depicted in Fig 5d. E.g. the spline suggests that predicted case fatality is lower at an incidence of 60 (the first split point) compared to an incidence of 160 (the final split point). With respect to the variable Medical doctors, the raw odds ratio for fatality above 1.5 doctors per capita compared to below is (1939 / 22156) / (792 / 6950) = 0.77 (with the CI entirely below 1), similar to the univariate analysis, which suggests that fatality generally declines with an increasing number of Medical doctors. But the spline relationship is very clearly showing the opposite effect. I understand that these are different modelling approaches, as the authors explained in their previous response, but these results are nonetheless *very* difficult to reconcile. E.g. the spline-predicted fatality above 1.5 doctors is higher at every point along its curve than the predicted fatality below 1.5 doctors. Given that the authors make conclusions about this relationship (580-584), I would strongly suggest digging a bit further to explain this pattern (possibly in a Supplementary Appendix), and I reiterate that the most important fix would be to add the FU-specific data point to the figures (this applies to all spline figures). I show an example of how to do this with R code attached as a pdf to my review (I hope this is available to the authors).
*We believe that in the previous questions, the criteria for the dichotomization and truncation of the variables were exhaustively presented, according to the results of the GAMM and not arbitrarily. For the example cited by the reviewer, "Emergency beds" and "FHP units", the model showed no significance for values ranges above 1000 emergency beds and above 600 FHP teams (see splines in Figs 5e, 5f), therefore, the exclusion of these ranges was mandatory to comply with the model's premises and was based on evidence. We have pointed out that the analyzes were truncated for the selection of the ranges of significant values, thus, the conclusions and inferences apply for these analyzed intervals. To make this condition completely clear, we have included this observation in a textual way in the conclusions.*

*Concerning to the possible antagonism between the results of the univariate analysis and the GAMM graphics, it is necessary to understand that the representations produced by GAMM serve exclusively to observe the shape and significance of the relationships between the variables and the outcome (linearity of the association). The mean effect itself is effectively measured by the analysis carried out later, univariate and adjusted (using GLMM). In the case of the number of doctors, the comparison presented in Table 4 is the proportion of fatal cases in FU with more 1.5doctors/1000 inhab versus fatal cases in FU with less than 1.5doctors/1000 inhab. The fact that FU with more than 1.5 doctors/1000 inhab have a lower proportion of deaths from VL does not prevent that, for FU with number up to 1.5 doctors/1000 inhab, the greater the number of medical doctors, the higher the VL case-fatality. One possible explanation is the number of medical doctors to work as a marker, and in this range of values (up to 1,5 medical doctors), there are other factors acting toward to outcome. In other words, apparently the number of medical doctors influences the outcome differently depending on the range of values. After a given value, the variable represents a protection marker. That is a perfectly plausible phenomenon. It is also necessary to consider that the adjustment at the FU level estimates the effect in average terms. There is also the possibility of an ecological fallacy.*

*Finally, the spines produced by GAMM do not allow the estimation of the beta effect, for this reason, they were not used for adjusted analyzes. Despite we have appreciated the reviewer's effort to produce the R code we were unable to produce the figure because the example sent consisted of only one explanatory variable, while all of our models are also adjusted by the year of notification.*

28. Relatedly, the univariate analysis suggests higher case fatality for males compared to females (Table 2) whereas the multiple analysis suggests substantially *lower* case fatality for males (Table 3). Again, I understand that these are different analyses, but this difference warrants further investigation and explanation, rather than just affirming the finding that female gender is "a known risk factor".

*We have included information about similar findings available in the literature and an explanation for the discrepancy between the univariate and adjusted models.*

29. The reference numbering looks to be off again in places. E.g. at line 199, the reference [26,27] should instead of [27,28].

*The references were checked and corrected.*

30. There is a Table 1 and 2 in the Methods, and another Table 1 and 2 in the Results. Normally tables are numbered sequentially through the entire manuscript.

*Tables were all numbered in ascending order.*

31. Between submissions #2 and #3, the units in the axis labels for Figs 5a and 5c changed from "days" to "weeks", but the axis values didn't change. Just confirming that this was intentional? Also, Fig. 5h (Garbage collection) changed in a way that I don't understand. The range of the x-values (1.6-2.8) doesn't correspond with the units in Table 2 (Results)

*We confirm that the unit of measure is “days” and it was inadvertently indicated as weeks. Figure 5 was produced again with the necessary corrections.*
